# Biomechanical study between percutaneous vertebroplasty combined with cement pedicle plasty improves vertebral biomechanical stability: A finite element analysis

**DOI:** 10.1186/s12891-024-07689-3

**Published:** 2024-07-29

**Authors:** Hongtao Li, Changming Xiao, Hongyu Pan, Yang Lei, Haozhong Wang, Sen Li

**Affiliations:** 1 Department of Spine Surgery, The Affiliated Traditional Chinese Medicine Hospital, Southwest Medical University, Luzhou, Sichuan Province, 646000, China; 2 Division of Spine Surgery, Department of Orthopedic Surgery, Affiliated Hospital of Medical School, Nanjing Drum Tower Hospital, Nanjing University, Nanjing, Jiangsu Province 210000 China

**Keywords:** Unstable osteoporotic vertebral fractures, Vertebroplasty, Pedicle plasty, Bone cement, Finite element analysis

## Abstract

**Objective:**

To investigate the biomechanical effects of percutaneous vertebroplasty combined with cement pedicle plasty (PVCPP) on the unstable osteoporotic vertebral fractures (OVFs) through finite element (FE) analysis. The study compares the biomechanical stability of finite element models between percutaneous vertebroplasty (PVP) and percutaneous vertebroplasty combined with cement pedicle plasty.

**Methods:**

Two patients with unstable OVFs underwent computed tomography (CT) examination at the thoracolumbar vertebral body levels, respectively. The CT images were reconstructed into three-dimensional finite element models to simulate stress conditions across six dimensions and to evaluate the vertebral von Mises stress before and after bone cement reinforcement.

**Results:**

The study found that stress distribution differed between groups mainly at the pedicle base. In the surgical vertebral bodies, the maximum stress in the PVP group decreased during flexion and left bending, while it increased in other states. In the PVCPP group, all maximum stresses decreased. In the inferior vertebral bodies, the maximum stress in the PVP group generally increased, while it decreased in the PVCPP group. In the superior vertebral bodies, postoperatively, the maximum stress in the PVP group generally increased, while it almost remained unchanged in the PVCPP group. PVP group had higher cement stress and displacement.

**Conclusion:**

PVCPP is an effective treatment method for patients with unstable OVFs. It can quickly relieve pain and enhance the stability of the three columns, thereby reducing the risk of some complications.

## Introduction

Osteoporotic vertebral fractures (OVFs) are a common complication of osteoporosis and are becoming increasingly prevalent due to population aging and nearly 90% of spinal fractures occur in the thoracolumbar region [1]. These may include unstable vertebral fractures with damage to the anterior, middle, and posterior columns and pedicles [2–4]. These patients are at higher risk for daily trauma, which, if left untreated, can lead to severe vertebral fractures that can lead to ongoing pain and even neurological symptoms.

Percutaneous vertebroplasty (PVP) is a minimally invasive surgery performed under local anesthesia, known for its quick recovery, significant therapeutic effects, and high acceptance rate. It is increasingly considered a standard surgical method for treating osteoporotic vertebral fractures [5]. The bone cement-strengthened area of PVP is mainly concentrated in the front 2/3 of the vertebral body, and the unfilled bone cement area in the back 1/3 of the vertebral body forms an area with relatively weak stiffness and strength [6]; however, it plays a vital role in stability and preventing vertebral body collapse and kyphosis. Unstable OVFs are usually accompanied by fractures of the posterior 1/3 of the vertebral body (including fractures of the central column, pedicle base, etc.). Some scholars believe that postoperative complications such as bone cement mass displacement, vertebral body collapse, and kyphosis may occur after PVP treatment for patients with such fractures, leading to the risk of recurrence of pain and even surgical failure [7, 8]. Therefore, traditional PVP treatment, such as vertebral fractures, has been controversial. As early as 2004, Eyheremendy et al. [9] reported using bone cement to fill the vertebral pedicles while performing percutaneous vertebroplasty to treat patients with osteoporotic vertebral fractures. Moreover, percutaneous vertebroplasty combined with cemented pedicle plasty has been used in the treatment of Kummell disease [10]. Compared with single PVP, the former shows better advantages in preventing the displacement of bone cement and postoperative follow-up [11]. In a preliminary clinical retrospective study, it has been previously reported in the literature that percutaneous vertebroplasty combined with cement pedicle plasty (PVCPP) was used to treat osteoporotic thoracolumbar burst fractures and achieved good clinical results [12]. However, at present, the scope of application of this surgical method is relatively limited, and adequate biomechanical verification still needs to be investigated.

The finite element (FE) model was considered to be helpful in biomechanical studies [13], and it can avoid the use of human specimens and minimize the variations due to inherent differences among individual parameters [14, 15]. To further understand the impact of PVCPP on vertebral bodies and bone cement, we reviewed the data of two patients undergoing PVP and PVCPP treatments for a single segment of unstable OVF, respectively. We constructed a vertebral biomechanical finite element model to preliminarily observe and discuss the biomechanical characteristics of both surgical methods, providing a theoretical basis for the rational application of the PVCPP technique.

## Materials and methods

### Patients with unstable OVFs

In this research, two female volunteers with unstable osteoporotic vertebral fractures (64 years old/68 years old, BMD: -2.6/-2.7) underwent CT scans at the levels of T10-T12 and T11-L1, respectively, both before and after undergoing treatment with PVP or PVCPP. These scans had a slice thickness of 0.625 mm. Subsequently, the CT images were reconstructed to create 3D FE models. On T1-weighted MRI, the affected vertebral body exhibited a low signal, while showing a high signal on T2-weighted MRI, ruling out other pathological fractures. All CT images were archived in the digital imaging and communications in medicine (DICOM) format. This study obtained an exemption from ethical approval from the Ethics Committee of the Affiliated Hospital of Traditional Chinese Medicine of Southwest Medical University due to the use of anonymized data. Informed consent from the patients was obtained.

### The construction of 3D models and fracture models

Two 3D models were developed based on the preoperative CT scans of T10-T12 and T12-L2 vertebral bodies, respectively. The CT images of 2 patients in DICOM format were imported into Mimics to generate the 3D model of the T10-T11-T12 and T12-L1-L2 vertebral bodies, respectively, including the cortical (1 mm thick) and cancellous bone. The vertebral bodies and intervertebral disc models use SOLID187 ten-node tetrahedral mesh element type. The following steps from the segmentation menu were performed: threshold segmentation was used to separate the bone and soft tissue, and editing mask tools were used to edit the image shape, select the desired area, fill the image area appearing in the gap, and split out the required contour layer by layer. Finally, 3D models were reconstructed through the edit mask option. The reconstructed 3D models were saved in STL format.

STL models were imported into the Geomagic automatic reverse engineering software for processes such as noise reduction, feature removal, and the application of structural patches and fitting surfaces. This resulted in the generation of the surface for 3D models of vertebral bodies, which were subsequently saved in STP format. Among them, Solidworks was used to run commands such as stretch, move, copy, and combine (delete) to create the intervertebral disc. A sketch was drawn on the upper and lower surfaces of the intervertebral disc to divide the intervertebral disc into the upper and lower endplates, and then divided the nucleus pulposus and annulus fibrosus. Optimization of these models was carried out using Geomagic Studio software before importing them into SolidWorks. There, the vertebral bodies were assembled, aligned, and manipulated using the surface command to create the endplates, cartilage, and intervertebral discs (comprising both the nucleus pulposus and annulus fibrosus). We used a linear spring element that only bears tension and not compression to simulate the solid ligaments, and the linear stiffness equation was as follows: F = kΔL. All specific anatomical data and corresponding material parameters for the ligaments are detailed in Table 1. Where, F is the force exerted by the ligament, k is the stiffness constant of the ligament, and ΔL is the deformation (displacement) of the ligament. The comprehensive 3D model was achieved by assembling these components. To simulate the vertebral fracture line, a previously established simulation method was employed, utilizing the surface command to incise the vertebral body, thus creating a 0.5-mm fracture line [16].

**Table 1:** Material properties of finite element analysis models

Component	Young modulus (MPa)	Poisson ratio	Element type	References
Normal cortical bone	12,000	0.3	SOLID187	[17]
Osteoporotic cortical bone	8040	0.3	SOLID187	[15]
Normal cancellous bone	132	0.2	SOLID187	[16]
Osteoporotic cancellous bone	34	0.2	SOLID187	[18]
Normal endplate	1000	0.4	SOLID187	[19]
Osteoporotic endplate	670	0.4	SOLID187	[20]
Intervertebral disc			SOLID187	[21]
Bone cement (PMMA)	3000	0.4	SOLID187	[22]
Nucleus pulposus	1	0.499		[23]
Annulus fibrosus	4.2	0.45		[22]
ALL	20	0.3	spring	[24]
PLL	20	0.3	spring	[23]
ISL	12	0.3	spring	[23]
SSL	15	0.3	spring	[23]
LF	19.5	0.3	spring	[23]
CL	7.5	0.3	spring	[23]
LI	12	0.3	spring	[23]

*ALL* anterior longitudinal ligament, *PLL* posterior longitudinal ligament, *ISL* interspinous ligament, *SSL* supraspinal ligament, *LF* ligamentum flavum, *CL* capsular ligament, *LI* ligamenta interspinalia, *PMMA*, polymethylmethacrylate

### Post-surgery augment models and Finite element analysis models

**Fig. 1:**
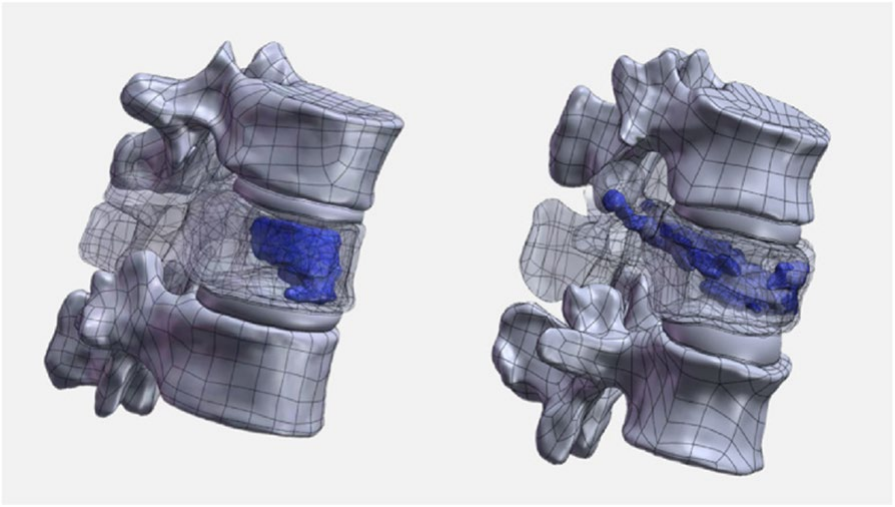
T10-T12, T12-L2 3D models and fracture models

**Fig. 2:**
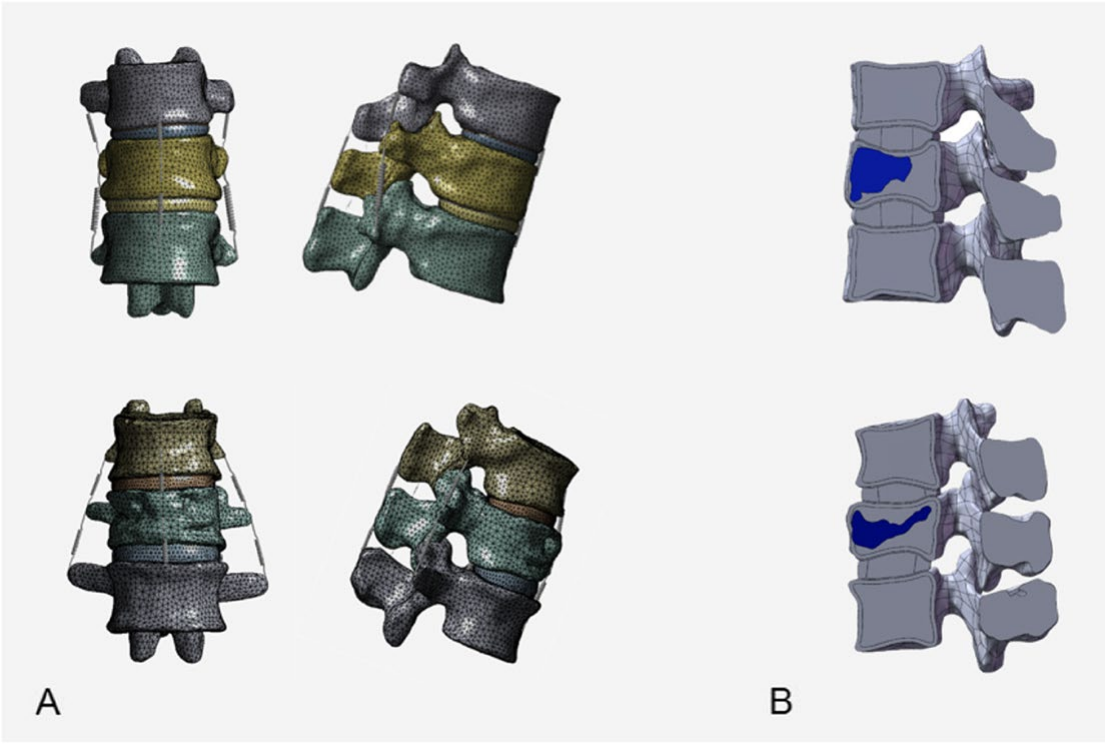
Finite element analysis models. **A** Finite element model of PVCPP group and PVP group. **B** Sagittal view of finite element models (showing cross-section of cortex and cancellous substance)

STP models were imported into SolidWorks for further processing. Extract and establish postoperative bone cement models from postoperative CT scans of vertebral bodies, each with a volume of 5 ml. In the T11 and L1 vertebral models, the bone cement was centrally positioned using the assembly feature. Subsequently, through the application of the software's Boolean operations, unnecessary bone material was excised, integrating the bone cement model seamlessly into the vertebral body, as illustrated in Fig. 1. This process yielded a 3D model of the T11 and L1 vertebral bodies post-bone cement augmentation. Table 1 presents the material properties utilized in recent studies concerning OVFs. FE Analysis was conducted using ANSYS software, wherein the 3D models—including those of the cortical and cancellous bones, bone cement, endplates, cartilage, and intervertebral discs (nucleus pulposus and annulus fibrosus)—were imported for comprehensive analysis. The contact between small joints is defined as facet-to-facet contact. The interfaces between vertebral bodies and endplates, endplates and intervertebral discs, and vertebral bodies and bone cement are all set to be fully bonded (Fig. 2).

### Force analysis of finite element model

**Fig. 3:**
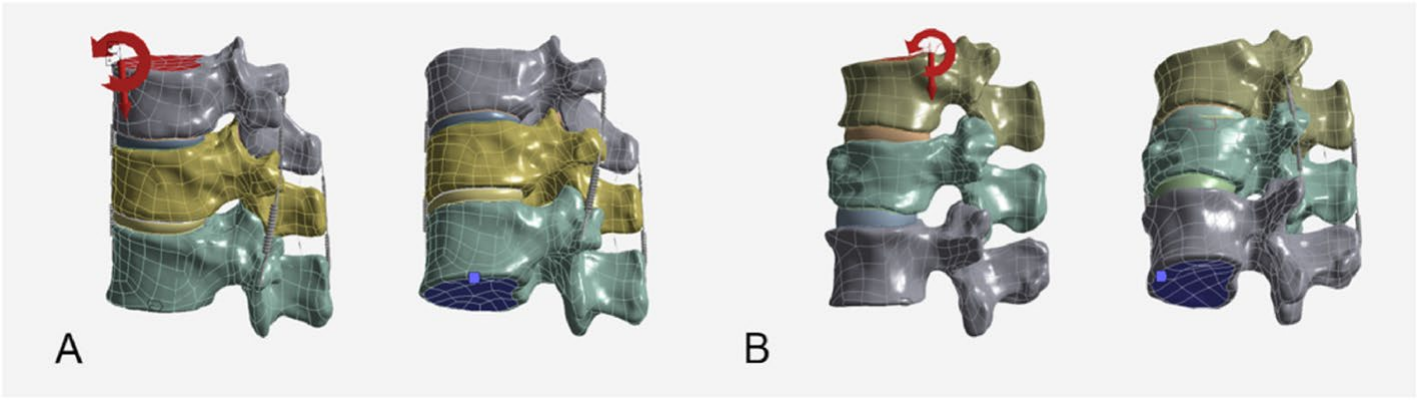
Boundary loading conditions for the finite element models. **A** Finite element model of PVP group. **B** Finite element model of PVCPP group

To constrain the boundary conditions of the thoracolumbar spine models after processing, all nodes and degrees of freedom in all directions on the lower surface of T12 and L1 vertebral bodies are fully constrained to make them fixed throughout the simulation process. Vertical downward compressive loads of 500N are applied respectively on the upper surfaces of T10 and T11 vertebral bodies to simulate the weight of the upper body, while 10 N^.^m torque is added on the upper surface of the superior vertebrae to achieve changes in the direction of model motion, simulating flexion, extension, lateral bending, and left/right axial rotation of the vertebrae (Fig. 3) [17, 25]. According to the three-column concept of the spine, loads and moments are applied to the upper endplate and articular surface of the T12 vertical body, with 85% of the loads and moments applied to the anterior-middle columns and 15% to the posterior columns [3, 26]. This study aims to assess the overall biomechanical changes of vertebral bodies after surgery using patient-specific CT scans. The equation of all von Mises stress calculation was as follows: a_=_ (((a1-a2)^2 + (a2-a3)^2 + (a3-a1)^2)/2)^0.5. Where, a is the maximum von Mises stress, and a1, a2, and a3 are the principal stresses in the three principal stress directions of the material. All vertebral bodies, bone cement stress results, and stress cloud diagrams obtained after the analysis can be exported to the computer. We calculated the overall stress of the surgical vertebral bodies, the adjacent vertebral bodies, and the bone cement. Due to the anatomical differences between the two patient groups, the final results are expressed as the percentage change in maximum stress before and after surgery to assess bone cement augmentation's effectiveness.

## Result

### Mesh convergence verification of finite element models

By varying the mesh size of the T12-L2 segment to create four different mesh resolutions (with mesh size differences not exceeding 25%), the maximum stress in the T12-L2 flexed position was compared. The mesh element sizes were 3.3mm, 2.5mm, 1.9mm, and 1.4mm, respectively. The results showed that for mesh sizes of 2.5mm and 1.4mm, the maximum stress values in T12-L2 were similar (the maximum difference did not exceed approximately 5%). However, further reducing the element size from 2.5mm to 1.9mm did not significantly change the peak stress, but greatly extended the solution time (Table 2). Similarly, such calculations were also performed in the motion direction of all finite element models. Therefore, a mesh density with an element size of 2.5mm was considered more reasonable.

**Table 2:** Mesh convergence verification of the finite element model

Mesh Size (mm)	Vertebral Body	Maximum Stress (MPa)	Percentage
3.3	T12	21.0	
	L1	24.6	
	L2	22.0	
2.5	T12	20.1	4.54%
	L1	25.9	5.58%
	L2	22.2	0.78%
1.9	T12	19.1	4.80%
	L1	25.7	0.77%
	L2	21.6	2.76%
1.4	T12	19.3	0.80%
	L1	24.6	4.46%
	L2	21.0	2.87%

### Calculation results of the maximum von Mises stress of the surgical vertebral bodies for PVP and PVCPP

**Fig. 4:**
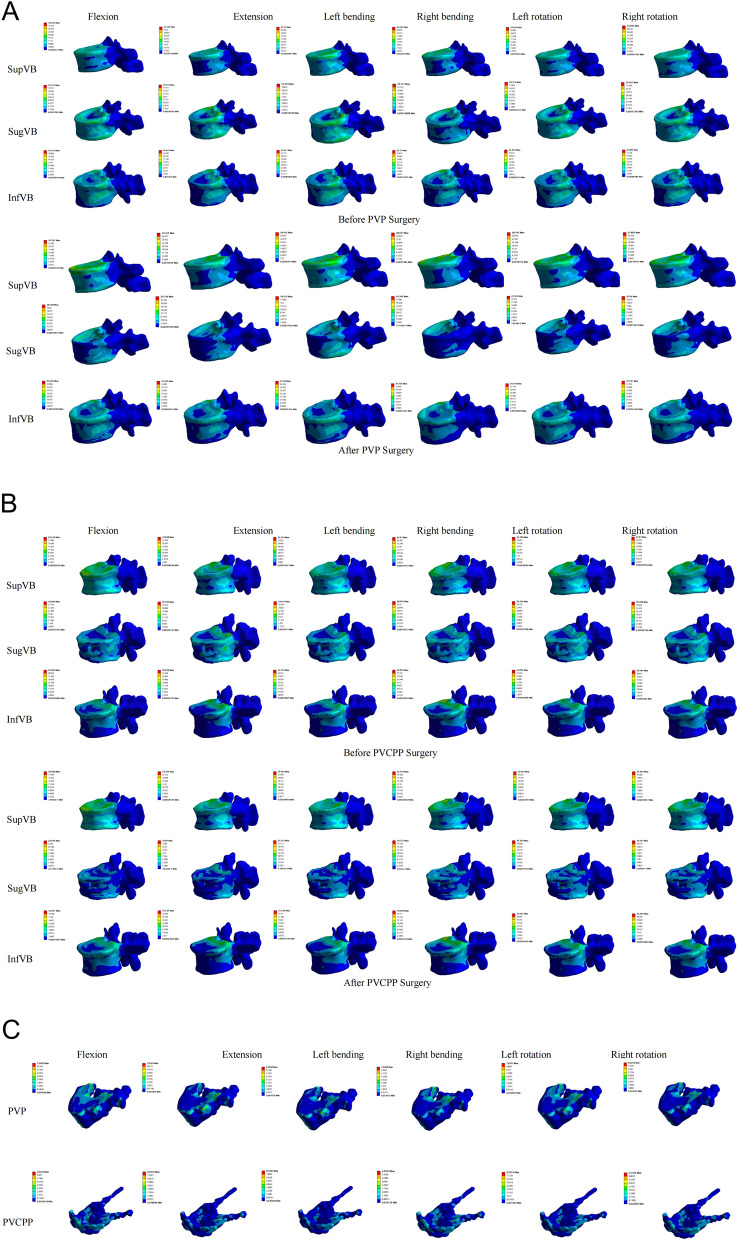
Stress cloud diagram under different working conditions of each group of models: **A** PVP; **B** PVCPP; **C** Bone cement

**Fig. 5:**
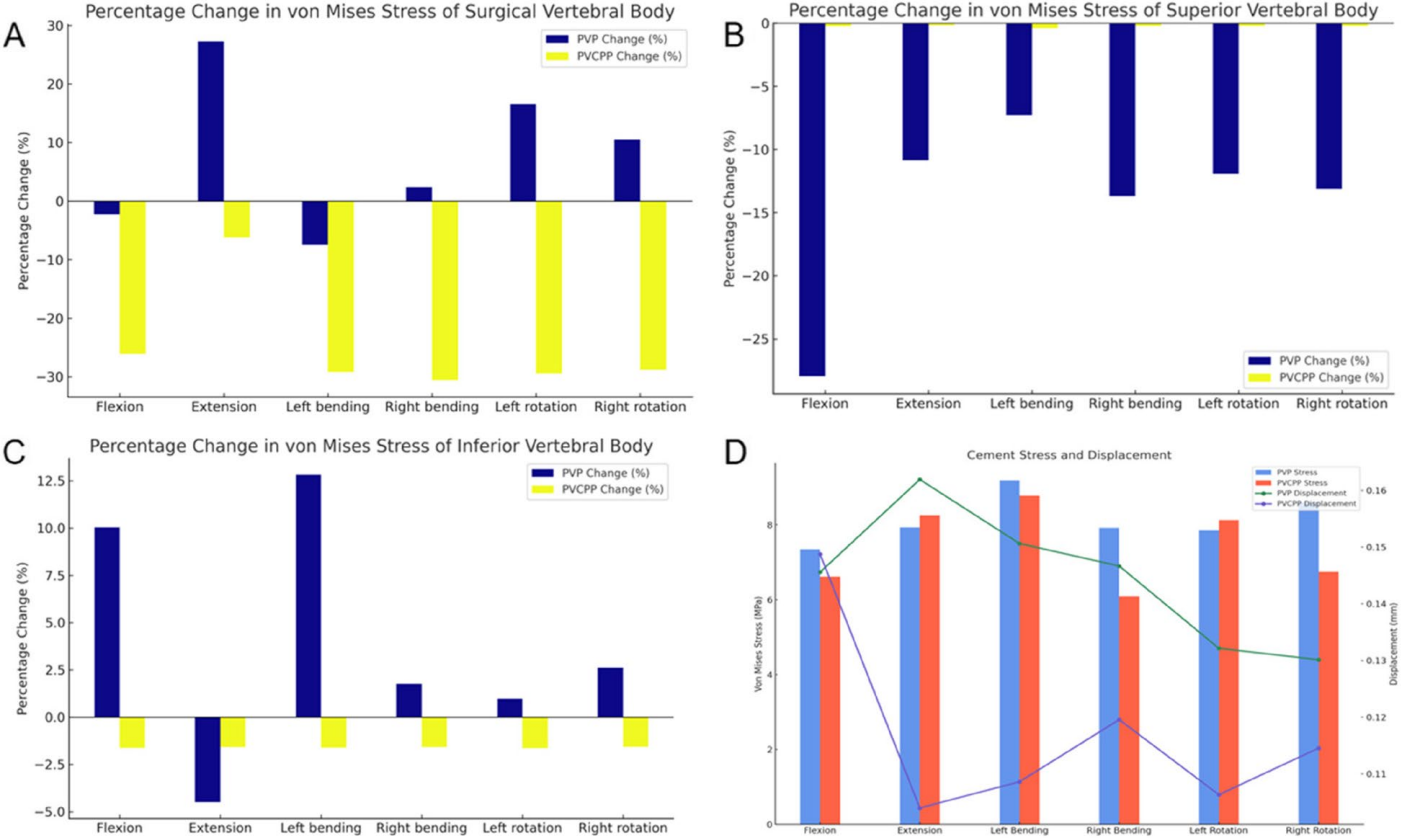
Statistical graphs of results under different experiments: **A**-**C** Statistical histogram of surgical, superior, and inferior vertebral stress experimental data for PVP and PVCPP (Percentage change). **D** Statistical graphs of results bone cement stress and displacement

The maximum von Mises stress cloud diagrams of the two groups of surgical vertebral bodies are shown in Fig. 4. The calculation results and statistical histograms after loading the same load are shown in Table 3 and Fig. 5A. The results indicated that under the conditions of forward flexion, extension, lateral bending, and rotation, the main difference in stress distribution between the two groups of patients' surgical vertebral bodies lies at the pedicle base in the PVCPP group. The postoperative maximum stress in the PVP group decreased during forward flexion and left lateral bending, while the stress increased in the remaining states. The postoperative maximum von Mises stress variation in the PVP group was -2.24%, + 27.29%, -7.47%, + 2.41%, + 16.60%, and + 10.47%. In contrast, the postoperative maximum von Mises stress in the PVCPP group decreased, with a maximum stress variation of -26.08%, -6.25%, -29.19%, -30.55%, -29.40%, and -28.8%.

**Table 3:** Calculation results of vertebral bodies von Mises stress (MPa) before and after PVP and PVCPP surgery (results were rounded to one decimal place)

	Flexion			Extension			Left bending			Right bending			Left rotation			Right rotation		
		Pre Post			Pre Post			Pre Post			Pre Post			Pre Post			Pre Post	
PVP SugVB	19.1 18.7			19.7 25.1			21.2 19.7			23.8 24.3			19.2 22.4			19.6 21.7		
PVCPP SugVB	35.1 25.9			26.5 24.8			33.5 23.8			28.4 19.7			30.1 21.3			30.4 21.6		
PVP SupVB	36.4 26.2			37.4 33.3			31.7 29.4			32.5 28.0			32.0 28.1			32.1 27.9		
PVCPP SupVB	20.1 20.1			23.8 23.8			24.3 24.2			22.2 22.1			22.1 22.1			22.0 21.9		
PVP InfVB	21.5 23.6			26.6 25.4			24.4 27.6			23.7 24.1			24.2 24.5			23.9 24.5		
PVCPP InfVB	22.6 22.2			25.0 24.6			27.6 27.1			20.0 19.7			24.0 23.6			23.6 23.2		

Pre, pre-augmented. Post, post-augmented. *PVP*, percutaneous vertebroplasty. *PVCPP*, percutaneous vertebroplasty combined with cement pedicle plasty. Sug*VB*, surgical vertebral body. Sup*VB*, superior vertebral body. In*fVB*, inferior vertebral body

### Calculation results of the maximum von Mises stress of the adjacent vertebral bodies for PVP and PVCPP

The maximum von Mises stress cloud diagrams of the two groups of adjacent vertebral bodies are shown in Fig. 4. The calculation results and statistical histograms after loading the same load are shown in Table 3 and Fig. 5B-C. Based on the calculations for the superior vertebral bodies, under the conditions of forward flexion, extension, lateral bending, and rotation, the postoperative stress distributions of the two finite element model groups showed little difference, and the maximum von Mises stress was reduced in both. For the superior vertebral body in the PVP group, the maximum stress variation was -27.95%, -10.87%, -7.29%, -13.71%, -11.93%, and -13.12%. In contrast, the PVCPP group showed a much smaller maximum stress variation in the superior vertebral body, with values of -0.24%, -0.18%, -0.40%, -0.22%, -0.20%, and -0.20%, respectively. Similarly, based on the calculations for the inferior vertebral bodies, the postoperative maximum von Mises stress generally increased in the PVP group, with stress changes of + 10.04%, -4.50%, + 12.82%, + 1.75%, + 0.97%, and + 2.62%. In the PVCPP group, the postoperative stress uniformly decreased, with changes of -1.62%, -1.26%, -1.61%, -1.58%, -1.64%, and -1.56%.

#### Calculated results of the experimental data of bone cement

Stress on the implant is one of the most important indices for evaluating its stability. A smaller maximum von Mises stress on the bone cement indicates a lower possibility of loosening and displacement of bone cement. The relative displacement of bone cement also reflects its stability. The distribution of the maximum von Mises stress for the two groups of bone cement is shown in Fig. 4C. The statistical histogram is presented in Fig. 5D. The calculation results showed that the bone cement in the PVP group exhibited higher maximum von Mises stress in flexion, left bending, right bending, and right rotation, with stress values of 7.3482 MPa, 9.1908 MPa, 7.9208 MPa, and 8.6053 MPa, respectively. This indicated that cement loosening or displacement risk in these four conditions was much higher than in the PVCPP group.

Furthermore, the stress distribution in the bone cement of the PVP group was uneven, suggesting that the local average stress on the bone cement was higher than that in the PVCPP group, increasing the probability of local complications. However, the PVCPP group exhibited higher maximum von Mises stress in extension and left rotation, with values of 8.2542 MPa and 8.1251 MPa, respectively. Overall, the bone cement in the PVP group is subject to higher average stress than that in the PVCPP group, indicating that the PVCPP group has more excellent stability.

The relative displacement of bone cement also reflects its stability. The smaller the relative displacement, the better the stability of the bone cement. In this experiment, the displacement of the bone cement in the vertebral bodies relative to its initial position was calculated. The calculation results and statistical histograms of the relative displacement of different groups of bone cement are shown in Fig. 5D. Under the same load. The PVP group exhibited poorer bone cement stability regarding extension, lateral bending, and rotation. Its cement displacement was the highest, with extension showing 0.16191 mm and 0.10397 mm, left bending showing 0.15062 mm and 0.10859 mm, right bending showing 0.14665 mm and 0.11955 mm, left rotation showing 0.13213 mm and 0.10631 mm, and right rotation showing 0.13011 mm and 0.11452 mm. In terms of flexion, the measurement results of the PVP and PVCPP groups were not significantly different. The evaluation results indicate that the PVP group has the lowest stability of bone cement, with the highest relative displacement.

## Discussion

In 1984, Galibert and others first applied PVP to the treatment of vertebral hemangiomas, achieving good clinical outcomes [27]. Over decades of development, this technique has become the gold standard for treating osteoporotic vertebral fractures clinically, effectively increasing vertebral bodies strength and stiffness, and quickly alleviating pain and restoring daily life [28]. However, a limitation exists where PVP mainly targets the anterior two-thirds of the vertebral body, leaving the posterior third unfilled with cement. Being a zone of relative weakness, this unfilled region is critical in ensuring vertebral stability and preventing deformities [6]. In PVP procedures, the "blind spot" in the posterior third of the vertebral body is often overlooked. Therefore, there is a controversy over its use in treating unstable OVFs. In 2002, Eyheremendy et al. [9] applied pedicle strengthening surgery to treat pedicle osteolytic lesions for the first time and achieved good clinical results. Later, in 2004, Van der Schaaf et al. [29] combined bilateral PVP with bilateral pedicle cement injection for treating Kummell's disease, effectively connecting the vertebral columns into a single structure with cement, yielding good results. They believed this method could effectively avoid the displacement of bone cement and prevent the posterior wall of the vertebral body from moving into the spinal canal later, causing spinal neurological symptoms. In the meantime, they speculate that bone cement pedicle plasty is the critical factor.

Percutaneous vertebroplasty combined with cement pedicle plasty was developed based on PVP. Bone cement is slowly injected into the first two-thirds of the vertebral body. Then, slowly retract the bone cement pusher and inject an appropriate amount of bone cement simultaneously. While the catheter is slowly withdrawn, sufficient bone cement is left in the pedicle. The three columns are gradually filled with bone cement through careful operation during the operation. It is worth noting that percutaneous vertebroplasty combined with bone cement pedicle plasty has been used to treat Kummell disease and pedicle osteolytic metastatic tumors and has achieved good clinical results [10, 30]. Theoretically, the advantages of this approach include providing mass effect, preventing vertebral bodies from collapsing, and supporting the three columns and pedicles to enhance structural stability. Moreover, this surgical method allows the bone cement of the vertebral body to be fixed in the vertebral body through the bone cement in the pedicle so that the bone cement is completely connected to the normal bone tissue, which can effectively prevent the bone cement from shifting. Such patients have poor vertebral body stability and severe osteoporosis. We perform routine surgery on these patients, and the postoperative prognosis is good. However, this surgical method's long-term clinical follow-up efficacy and biomechanical stability analysis are currently needed.

The FE method has proven to be effective in simulating spine motion thanks to its predictive solid ability [31, 32]. It offers the advantage of low cost for numerical experiments and allows for adjusting various parameters, including material properties [33]. Furthermore, the model can provide results that are difficult to obtain through invasive procedures [34]. By conducting FE analysis, it is possible to accurately simulate the anatomical dynamics of the human spine and observe biomechanical changes that closely resemble natural anatomical conditions [34, 35]. Early studies indicated that PVP altered the biomechanical strength of fractured vertebral bodies but could increase the risk of fractures in adjacent vertebral bodies [36]. Most literature reports on lumbar 3D finite element models are based on bony structures without considering the spine's posterior elements. Therefore, there may be inaccuracies in assessing the impact on the stress of vertebral bodies after traditional PVP surgery, which has limitations. This study is the first to explore finite element biomechanics after PVCPP treatment for unstable OVFs, using real pre- and postoperative CT data for 3D biomechanical finite element modeling and simulating the characteristics of osteoporotic vertebral material based on the physical properties of different tissues. This approach brings the 3D finite element model closer to the biomechanical properties of the vertebral bodies, more accurately reflects postoperative spinal biomechanical parameters, and detects stress changes in each vertebra more precisely after PVCPP treatment [37].

Our study found that under conditions such as flexion, extension, lateral bending, and rotation, the main difference in stress distribution between the patients’ surgical vertebral bodies lies at the pedicle base in the PVCPP group. Post-PVP, the maximum von Mises stress decreased during flexion and left bending, while it increased in other states. In contrast, post-PVCPP, the maximum von Mises stress decreased in all conditions. In the same six dimensions, the stress distribution on the superior vertebral bodies between the two finite element model groups showed little difference, with a decrease in maximum von Mises stress. Similarly, calculations for the inferior vertebral bodies indicated a general increase in maximum von Mises stress post-PVP, while in the PVCPP group, stress in the inferior vertebral body decreased. Therefore, the PVCPP group experienced more uniform stress distribution on the adjacent vertebral body without increasing the risk of fractures. Since vertebral fractures are closely related to the distribution and magnitude of stress, this technique can reduce the risk of fractures in adjacent vertebral bodies [38]. Moreover, bone cement's stress and relative displacement are essential to its stability. The smaller the maximum von Mises stress on the bone cement, the less likely it is to loosen and displace. In this experiment, the average stress on the bone cement in the PVP group was higher than in the PVCPP group, indicating more substantial stability in the PVCPP group. Moreover, the relative displacement of bone cement was highest in the PVP group. Therefore, the vertebral body's overall stability is more substantial after bone cement pedicle augmentation. The observed results are primarily due to the advantages of PVCPP: Firstly, bilateral pedicle approach puncture allows for a more uniform distribution of bone cement, as previously reported [39]. Additionally, beyond the anterior two-thirds of the vertebral body filled in traditional vertebroplasty, bone cement also fills the weaker posterior third's “blind spot” and the interior of the pedicle, leading to more uniform stress distribution on the vertebral bodies. The "bone cement screw" effect effectively integrates the fracture area, preventing displacement of fracture fragments and cement, and further effectively prevents uneven stress distribution caused by kyphotic deformity leading to sagittal imbalance.

There are some limitations for this research. Firstly, The models in this study were all based on patient-specific CT data modeling. The finite element model established before and after surgery was a pathological model. The model was not compared with the normal model to verify its effectiveness. Secondly, the research did not use specific parameters for the intervertebral disc proposed by Niemeyer et al. [40], 2012 for precise modeling and the callus behaves as a poroelastic medium, and studying stress, as well as fluid entry into the callus, is necessary to reduce the efficiency of the ossification process [41]. Thirdly, this study focuses on the overall biomechanics of the skeleton, involving preliminary macroscopic analysis, without considering the influence of bone density and skeletal heterogeneity [42]. Fourthly, because the clinical manifestations and imaging of the two patients did not involve disc herniation, intervertebral disc protrusion related to pain was not considered [43]. Fifthly, static loading does not fully simulate the dynamic kinematics of human activities throughout the day [44]. Lastly, this study is based on individual-specific CT data, and the same results found in this paper cannot be guaranteed to be applicable to the shape of any patient's skeleton [45, 46].

In summary, PVCPP is a minimally invasive, rapidly recovering, and effective surgical method that distributes the load evenly across the surgical and adjacent vertebral bodies and reduces stress. It is concluded that this technique significantly reduces the risk of vertebral fractures and prevents cement displacement, providing a reference for the clinical adoption of PVCPP in treating unstable OVFs. Although similar to vertebroplasty, the surgical risk increases due to cement injection near the spinal cord and nerve structures. Therefore, high-quality fluoroscopic guidance and high-viscosity bone cement are vital to this technique.

## Conclusion

Based on the results of finite element analysis experiments and previous clinical effects, percutaneous vertebroplasty combined with bone cement pedicle plasty is an effective treatment method for patients with this type of unstable OVFs. This surgical approach offers rapid pain relief and enhances the stability of the three columns, thereby reducing the chances of vertebral bodies refracture and preventing the intrusion of posterior wall fracture fragments into the spinal canal, which could damage the spinal nerves.

## Data Availability

All data generated or analyzed during this study are included in this published article.

## Abbreviations

ALL: Anterior longitudinal ligament
PLL: Posterior longitudinal ligament
ISL: Interspinous ligament
SSL: Supraspinal ligament
LF: Ligamentum flavum
CL: Capsular ligament
LI: Ligamenta interspinalia
Pre: Pre-augmented
Post: Post-augmented
PVP: Percutaneous vertebroplasty
PVCPP: Percutaneous vertebroplasty combined with cement pedicle plasty
SugVB: Surgical vertebral body
SupVB: Superior vertebral body
InfVB: Inferior vertebral body
OVFs: Osteoporotic vertebral fractures
FE: Finite element
DICOM: Digital imaging and communications in medicine
T3D2: Truss-3D-2node
3D: Three-dimension
